# Population structure and antimicrobial resistance of Corynebacterium diphtheriae in Victoria, Australia

**DOI:** 10.1099/mgen.0.001517

**Published:** 2025-10-01

**Authors:** Lamali Sadeesh Kumar, Sarah L. Baines, Kylie Hui, Janet Strachan, Norelle L. Sherry, Benjamin P. Howden

**Affiliations:** 1 Department of Microbiology and Immunology, The University of Melbourne at the Peter Doherty Institute for Infection and Immunity, Melbourne, Victoria, Australia; 2 Microbiological Diagnostic Unit Public Health Laboratory, Department of Microbiology and Immunology, The University of Melbourne at the Peter Doherty Institute for Infection and Immunity, Melbourne, Victoria, Australia; 3 WHO Collaborating Centre for Antimicrobial Resistance, The Peter Doherty Institute for Infection and Immunity, Melbourne, Victoria, Australia; 4 Centre for Pathogen Genomics, The University of Melbourne, Melbourne, Victoria, Australia; 5 Communicable Diseases, Community and Public Health, Department of Health, Melbourne, Victoria, Australia; 6 Department of Infectious Diseases & Immunology, Austin Health, Heidelberg, Victoria, Australia

**Keywords:** antimicrobial resistance, *Corynebacterium diphtheriae*, genomics, population structure, public health

## Abstract

*Corynebacterium diphtheriae*, the main aetiological agent of diphtheria, is a re-emerging bacterial pathogen of public health concern, yet remains largely understudied globally. In this study, we analysed the population structure and antimicrobial resistance (AMR) of 210 *C*. *diphtheriae* isolates from Victoria, Australia, including 103 historical (1950–1970) and 107 contemporary clinical isolates (2004–2023), using whole-genome sequencing and phenotypic susceptibility testing. The diphtheria toxin gene (*tox*) was detected in 89 isolates, the majority of which (*n*=83; 93.3%) were historical. Population structure comprised two primary phylogenetic lineages, Mitis and Gravis, each containing multiple sublineages. Multi-locus sequence type (MLST) analysis revealed a highly diverse population structure with multiple novel MLST profiles and alleles. When placed within a global phylogenetic framework, Australian isolates were broadly distributed, reflecting substantial genetic diversity. Phenotypic susceptibility testing against 11 antimicrobials revealed that several contemporary isolates were resistant to multiple agents, including penicillin and erythromycin, first-line treatments of *Corynebacterium* infections. Eight contemporary isolates were multidrug-resistant (resistant to ≥3 antimicrobial classes), including five with resistance to both penicillin and erythromycin. Genomic analysis identified multiple genes and mutations conferring resistance among contemporary isolates. In contrast, no AMR phenotypes or genotypes were observed in historical isolates. Analysis of historical genomes provides valuable insights into a period of heightened diphtheria activity in Victoria prior to widespread immunization. Overall, these findings establish a baseline for ongoing genomic surveillance in the face of increasing global outbreaks, support informed empiric treatment strategies and contribute to the knowledge of the global population structure of *C. diphtheriae*.

**Impact Statement**

*Corynebacterium diphtheriae* is a re-emerging pathogen of public health concern with limited genomic data available to support surveillance and public health interventions. This study provides a contemporary understanding of *C. diphtheriae* from Australia in the era of genomic surveillance and helps us better understand baseline genomic diversity and antimicrobial resistance. Additionally, these findings enhance preparedness for potential future incursions or outbreaks, including those involving drug-resistant strains, as recently observed in parts of Africa and Europe.

## Data Summary

Genome sequences are deposited in GenBank under BioProject PRJNA870170. Sample data and accession numbers are included in Table S1. The authors confirm all supporting data, code and protocols have been provided within the article or through supplementary data files.

## Introduction

*Corynebacterium diphtheriae* is a Gram-positive rod and the main aetiological agent of diphtheria when toxigenic. Toxigenicity occurs when the *C. diphtheriae* (or other related *Corynebacterium* species) is lysogenized by corynebacteriophages that carry the diphtheria toxin gene (*tox*) [1]. Diphtheria is a vaccine-preventable disease that can cause respiratory or cutaneous illness, with transmission occurring via respiratory droplets or through contact with infected ulcers. It was a major public concern in Australia with high morbidity and mortality rates in the early 1900s prior to the introduction of the diphtheria toxoid vaccine. However, nationwide immunization programmes have resulted in a dramatic decline of diphtheria cases in Australia and many other countries [2,4]. Nowadays, diphtheria is rarely reported in Australia, with a small number of sporadic cases, mostly acquired from outside of Australia [5].

Immunization against diphtheria provides protection only against toxigenic *C. diphtheriae* strains. However, multiple illnesses, including invasive diseases such as bacteraemia, endocarditis and septic arthritis caused by non-toxigenic strains, have been reported worldwide [6,9], with increasing incidence of disease caused by non-toxigenic strains reported from multiple world regions [10,12]. Widespread use of improved bacterial identification methods such as MALDI-TOF MS systems has enhanced the detection of *tox*-negative strains in the last decade [13,14]. In addition to toxigenic and non-toxigenic strains, non-toxigenic toxin gene-bearing (NTTB) *C. diphtheriae* strains have also been reported from multiple countries, including Australia [15,17]. By definition, NTTB strains are genetically positive for the diphtheria toxin gene, yet phenotypically negative for toxin production. Previous studies have discussed the potential role of NTTB strains as toxin gene reservoirs in the population with the theoretical possibility of re-emerging toxin expression [16,18]. However, such events have not been reported. While inactivating mutations in the toxin gene may explain the lack of toxin production in some NTTB strains, no definitive genetic explanation has been determined in others [17,18].

The primary treatment for diphtheria (other than supportive measures) is administration of diphtheria antitoxin to neutralize unbound toxin and reduce adverse systemic effects of diphtheria toxin. Antimicrobials are prescribed for all *C. diphtheriae* infections regardless of toxigenicity and recommended first-line antimicrobials include penicillin and erythromycin. Additionally, close contacts of a patient with confirmed diphtheria are prescribed antimicrobial therapy as chemoprophylaxis, along with primary or booster vaccination against diphtheria if necessary [1,17, 19]. However, reduced susceptibility or resistance to first-line antimicrobials and other antimicrobial agents, including multidrug resistance, has been reported globally [17,20,24]. Multiple genes and mutations have been described in *C. diphtheriae* conferring antimicrobial resistance (AMR), including the *pbp2m* gene [17] and *erm(X*) gene [25], conferring resistance to penicillin and erythromycin, respectively. Mobile genetic elements (MGEs), such as plasmids and integrons, have been reported to harbour AMR genes and enable their transfer between strains via horizontal gene transfer [17,25, 26].

Recently, *C. diphtheriae* has gained attention as a re-emerging pathogen causing multiple diphtheria outbreaks globally [24,27], including strains noted to exhibit significant AMR. Although toxigenic *C. diphtheriae* strains remain rare in Australia, non-toxigenic strains are regularly identified [5,9, 14]. Thus, it is important to establish and maintain surveillance of *C. diphtheriae* strains in Australia for outbreak detection and response and to monitor AMR.

Here we present a genomic portrait of *C. diphtheriae* recovered from the state of Victoria, Australia, over contemporary (2004–2023) and historical (1950–1970) years. This study aims to provide critical insights into the prevalence of the diphtheria toxin gene, phenotypic antimicrobial susceptibility profiles and associated genetic determinants of AMR, as well as to investigate temporal trends in these clinically relevant features. Additionally, we aim to establish a phylogenetic framework for understanding the local population structure of Victorian *C. diphtheriae* within a global context, thereby enhancing our understanding of the pathogen’s epidemiology in preparation for future incursions and outbreaks.

## Methods

### Study design and dataset

The study was performed at the Microbiological Diagnostic Unit Public Health Laboratory (MDU PHL) in the state of Victoria, Australia (population 7.0 million at 30 September 2024) [28], the laboratory responsible for statewide surveillance of bacterial public health pathogens for over 125 years, with an extensive collection of historical isolates. A total of 210 *C. diphtheriae* isolates were included in this study (Table S1, available in the online Supplementary Material). The dataset was divided into two subsets: contemporary clinical isolates (*n*=107) submitted by diagnostic laboratories in Victoria between 1 January 2004 and 31 December 2023, and a selection of historical isolates (*n*=103) submitted between 1950 and 1970. Duplicate contemporary isolates were identified and excluded, with the earliest isolate for each patient being included in the study. A subset of historical isolates preserved as freeze-dried material was randomly selected to represent the period 1950–1970, with only viable *C. diphtheriae* isolates included in the study. For historical isolates (*n*=103), available metadata were limited, comprising only isolate ID, patient name, year (or year range) of isolation and region of isolation, and those data were used to minimize duplication. All isolates were identified as *C. diphtheriae* by MALDI-TOF MS (VITEK^®^ MS) technology.

### DNA extraction, whole-genome sequencing and genome analysis

*C. diphtheriae* cultures were grown aerobically at 37 °C for 24 h on horse blood agar. Genomic DNA extraction was performed using the Virus/Pathogen DSP mini kit on the QIAsymphony (QIAGEN). DNA libraries were prepared using the Nextera XT kit following the manufacturer’s instructions (Illumina). Whole-genome sequencing was performed on the Illumina NextSeq 500/550 systems using 2×150 bp paired-end chemistry following the manufacturer’s instructions.

The Bohra bioinformatics pipeline (v2.3.7) (https://github.com/MDU-PHL/bohra) was used for quality control of sequence reads and genome assembly. SKESA (v2.5.1) (https://github.com/ncbi/SKESA) [29] was used as the *de novo* sequence assembler, run with default parameters. *Corynebacterium belfantii* and *Corynebacterium rouxii* strains, formerly classified as *C. diphtheriae*, were identified by genome-wide average nucleotide identity (ANI) on genomic sequence data as previously described [30] and were subsequently excluded from the study.

*In silico* multi-locus sequence typing (MLST) was performed with mlst (v2.23.0) (https://github.com/tseemann/mlst) following the MLST scheme [31] hosted at the Institut Pasteur (https://bigsdb.pasteur.fr/diphtheria/). Upon submission of all genome assemblies to the BIGSdb platform (Institut Pasteur), new sequence types (STs) were assigned to novel MLST profiles identified in this study.

### Detection of diphtheria toxin gene (*tox*)

Assembled sequences were screened for *tox* with AMRFinderPlus (v3.12.8). Additionally, *tox* detection for the subset of contemporary isolates was performed routinely at MDU PHL via a conventional PCR assay at the time of sample receipt [32]. Aligned *tox* sequences from assemblies against the *tox* sequence from the reference strain NCTC 13129 (NC_002935.2) were obtained using gene-puller (identity >95% and coverage >90%) and visualized with Geneious Prime (version 2024.0.7) (http://www.geneious.com) to investigate any genotypes related to NTTB strains as described previously [17,18].

### Victorian phylogenetic structure of *C. diphtheriae*

A whole-genome alignment, from mapping reads against reference genome *C. diphtheriae* NCTC 13129 (NC_002935.2), was generated for Victorian *C. diphtheriae* isolates using Snippy v4.6.0 (https://github.com/tseemann/snippy). An initial maximum likelihood (ML) tree was inferred with IQ-TREE 2 with the best-fit model TVM+F+I+G4 [33]. Recombination events were identified and the recombination-adjusted ML tree was inferred with ClonalFrameML (v1.13) [34] based on the whole-genome alignment. The phylogenetic tree was visualized and annotated with Interactive Tree of Life (iTOL) (v6) [35].

Previous studies have defined two main phylogenetic lineages, Mitis and Gravis, after observing a strong correlation with the presence (Gravis) or absence (Mitis) of the *spuA* gene [17]. Hence, the presence of *spuA* was investigated in genome assemblies using fastablasta (identity >90% and coverage >95%) (https://github.com/kwongj/fastablasta).

Pairwise SNP distances were calculated from the whole-genome alignment of the Victorian isolates using snp-dists (v0.8.2) (https://github.com/tseemann/snp-dists). Pairwise SNP distances for the ten most common STs were filtered and only isolates with pairwise distances of <100 SNPs within each ST were included in the subsequent analysis to identify closely related isolate pairs or clusters.

### Global phylogenetic structure of *C. diphtheriae*

The global population structure of *C. diphtheriae* was analysed using all 210 strains from this study alongside 885 publicly available strains (Table S2). Publicly available *C. diphtheriae* strains, derived exclusively from human clinical samples, were stratified by geography (continent) and year of isolation, then randomly subsampled to minimize overrepresentation of outbreak-related clusters. Of the 1,095 isolates, those collected between 2004 and 2023 were classified as contemporary (*n*=823), while those collected prior to 2004 were classified as historical (*n*=62). A phylogenetic tree for global population structure was created using mashtree (https://github.com/lskatz/mashtree) (--sketch-size 50,000 --seed 100 --min-depth 0). The mashtree was visualized and annotated with iTOL (v6) [35].

### Antimicrobial susceptibility testing

Phenotypic antimicrobial susceptibility testing (AST) was performed by broth microdilution (BMD) for the following 11 antimicrobials: ciprofloxacin, clindamycin, erythromycin, gentamicin, linezolid, penicillin, quinupristin/dalfopristin, rifampicin, tetracycline, trimethoprim/sulfamethoxazole and vancomycin. BMD was performed with direct colony suspensions equivalent to a 0.5 McFarland standard incubated in cation-adjusted Mueller–Hinton broth with lysed horse blood (2.5–5% v/v) at 35 °C in ambient air for 24 h. Sensititre AIM^™^ Automated Inoculation Delivery System was used for inoculating the broth to GPN3F Sensititre standard plates (Thermo Fisher Scientific, Waltham, MA, USA). Following incubation, minimum inhibitory concentration (MIC) values were read using the Sensititre ARIS HiQ System or Sensititre Vizion Digital MIC Viewing System (Thermo Fisher Scientific, Waltham, MA, USA). Quality control was performed with the *Streptococcus pneumoniae* ATCC^®^ 49619 reference strain. MIC values for the above antimicrobials were interpreted following the breakpoints for *Corynebacterium* spp. from Clinical and Laboratory Standards Institute (CLSI) M45 guidelines, third ed. [36]. *C. diphtheriae* strains that exhibited resistance to three or more antimicrobial classes were categorized as multidrug-resistant (MDR) *C. diphtheriae*.

### Detection of AMR determinants and associated MGEs

Known AMR mechanisms, including acquired AMR genes and *C. diphtheriae*-specific mutational resistance, were identified by analysing the genomic assemblies using AMRFinderPlus (v3.12.8) with *C. diphtheriae* as the species option. AMR genes with >90% coverage and >95% identity compared to the AMRFinderPlus database and without internal stop codons were retained for downstream analysis. Isolates with AMR genes were subsequently screened for the presence of MGEs – plasmids and integrons. Plasmids were predicted from genome assemblies using MOB-suite (v3.1.8) (https://github.com/phac-nml/mob-suite) [37], and plasmid-associated AMR genes were identified by using AMRFinderPlus (v3.12.8) to screen the MOB-suite predicted plasmid sequences. Similarly, integrons were first predicted with IntegronFinder (v2.0.1) (https://github.com/gem-pasteur/Integron_Finder) [38], and then those sequences were screened for AMR genes using AMRFinderPlus (v3.12.8).

## Results

### Characteristics of *C. diphtheriae* isolates in Victoria

All 210 isolates of this study were confirmed as *C. diphtheriae* with ANI values above 97% when compared with *C. diphtheriae* type strain NCTC11397^T^, thereby excluding *C. belfantii* and *C. rouxii*. Contemporary *C. diphtheriae* isolates (*n*=107) were collected from a range of clinical sample types, including cutaneous (*n*=77), respiratory (*n*=14), blood (*n*=3), other sources such as tissue, pus and ear swabs (*n*=8) and five isolates of unknown source (Table S1 and Fig. S1).

A total of 89 (42.4%) *C*. *diphtheriae* genomes were found to carry the *tox*, while the remaining 121 (57.6%) isolates were *tox*-negative. The *tox* was detected in 80.6% (83/103) of historical isolates and only 5.6% (6/107) of contemporary isolates (Fig. S1). PCR and *in silico* genomic detection of *tox* were 100% concordant for *tox*-positive contemporary isolates. Among all *tox*-positive isolates, the toxin gene remained intact, with no internal stop codons or other disruptive genetic events observed, such as those reported in NTTB strains.

For *tox*-positive historical isolates (*n*=83), available metadata were insufficient to assess potential epidemiological links between cases. The *tox*-positive contemporary isolates were reported in 2013 (*n*=2), 2017 (*n*=2), 2019 (*n*=1) and 2022 (*n*=1), with no identified epidemiological links between the cases. However, all six patients had a recent history of overseas travel, suggesting that these strains were likely imported (Table S1).

### Victorian *C. diphtheriae* isolates were highly diverse

**Fig. 1.**
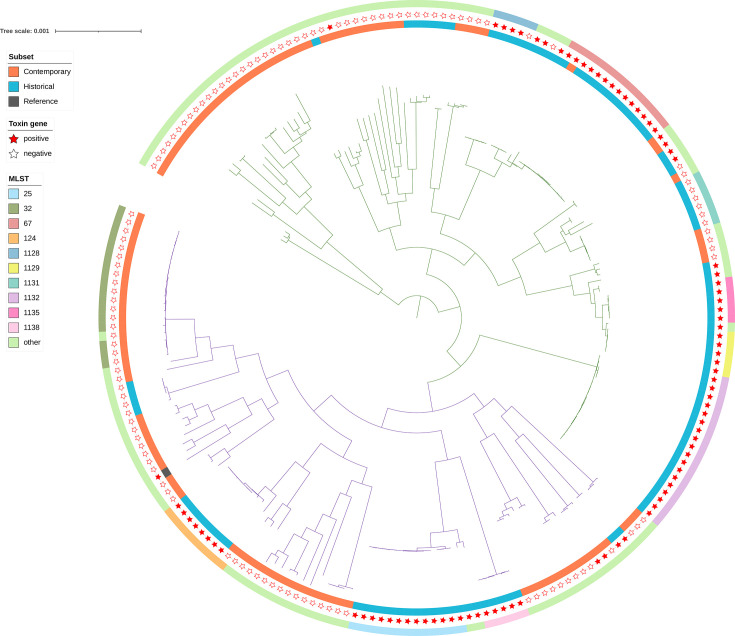
Phylogenetic tree (maximum likelihood, recombination adjusted) of 210 *C. diphtheriae* isolates from Victoria, Australia. The tree is constructed from a whole-genome SNP alignment relative to the reference genome *C. diphtheriae* NCTC 13129 (NC_002935.2). The root was set based on the divergence point of *C. belfantii* FRC0043a (not shown), and the branches of the main phylogenetic lineages, Mitis and Gravis, are coloured in green and purple, respectively. The inner ring corresponds to contemporary (*n*=107, orange) and historical (*n*=103, blue) isolates and reference genome (grey). The middle ring denotes the presence (filled star) or absence (unfilled star) of diphtheria toxin gene. The outer ring shows the ten most common STs and all remaining STs grouped as ‘other STs’.

MLST identified both known and novel STs among historical and contemporary isolates. Submission of novel profiles to the BIGSdb platform (Institut Pasteur) led to the assignment of 33 new STs across 79 isolates (22 contemporary, 57 historical). Contemporary isolates showed significant diversity, with 66 different STs represented, while historical isolates were represented by 21 STs (Table S1). ST32 was the most frequently reported ST among contemporary isolates (*n*=17), and it was exclusive to the contemporary subset. All isolates of ST32 were *tox*-negative and detected across multiple years between 2008 and 2023, with 12 of 17 isolated from respiratory samples. In comparison, historical isolates showed a higher prevalence of ST25 (*n*=13), ST67 (*n*=13) and ST1132 (*n*=18), all of which were *tox*-positive (Fig. 1 and Table S1). Four STs, ST67, ST124, ST20 and ST1134, were observed in both historical and contemporary subsets. Isolates of ST67 were all *tox*-positive and isolates of ST1134 were all *tox*-negative in both historical and contemporary subsets. In contrast, historical isolates of ST124 and ST20 were all *tox-*positive, while contemporary isolates of the same STs were *tox*-negative. Both *tox*-positive and *tox*-negative isolates were observed among historical isolates of ST1128 and ST1133 and among contemporary isolates of ST120 (Table S1).

Analysis of the pairwise SNP distances between isolates belonging to the ten most common STs revealed multiple putative clusters of closely related isolates (Fig. S2). Isolates from ST25, ST67, ST124, ST1128, ST1129 and ST1131 predominantly clustered within fewer than 40 SNPs. A bimodal distribution was observed for ST1138, with isolates differing either by 6–11 SNPs or 33–49 SNPs. In contrast, ST32, ST1132 and ST1135 displayed a wider range of pairwise SNP distances in the 0–100 SNPs distribution range. Isolates clustered within fewer than 40 SNPs were predominantly historic, *tox*-positive isolates. However, both *tox*-positive and *tox*-negative isolates were observed with fewer than 37 SNPs in ST1128. Among these ten STs, ST32 was the only contemporary ST with closely related isolates (<100 SNPs), reported over multiple years between 2008 and 2023 (Fig. S2 and Table S1).

Phylogeny of *C. diphtheriae* strains from Victoria rooted based on the divergence point of *C. belfantii* exhibited a star-like phylogeny with multiple deep branching clades (Fig. 1). As per ClonalFrameML (v1.13) results, the relative rate of recombination to mutation (*R*/theta) was 0.70. The average length of recombination segments (delta) was 649 bp and the mean genetic distance between donor and recipient of recombination (nu) was 0.02. This resulted in a relative impact of recombination to mutation (*r*/*m*=*R*/theta×delta×nu) of 7.5, which means that the homologous recombination impacts the Victorian *C. diphtheriae* diversification 7.5 times more than mutations.

The distribution of the *spuA* gene largely distinguished the two previously described phylogenetic lineages, Mitis and Gravis. Both lineages were equally represented among Victorian *C. diphtheriae* isolates. All but ten Gravis lineage isolates were *spuA*-positive, and all but two Mitis lineage isolates were *spuA*-negative. Both the Mitis and Gravis lineages were represented in both historical and contemporary isolates. *Tox*-positive and *tox*-negative isolates were found in both the Mitis and Gravis lineages; however, a higher proportion of *tox*-positive isolates was observed in the Mitis lineage (57/115; 50%) compared to the Gravis lineage (32/95; 33.7%) (Table S1 and Fig. 1).

### Australian isolates were widely dispersed throughout the global phylogeny

**Fig. 2.**
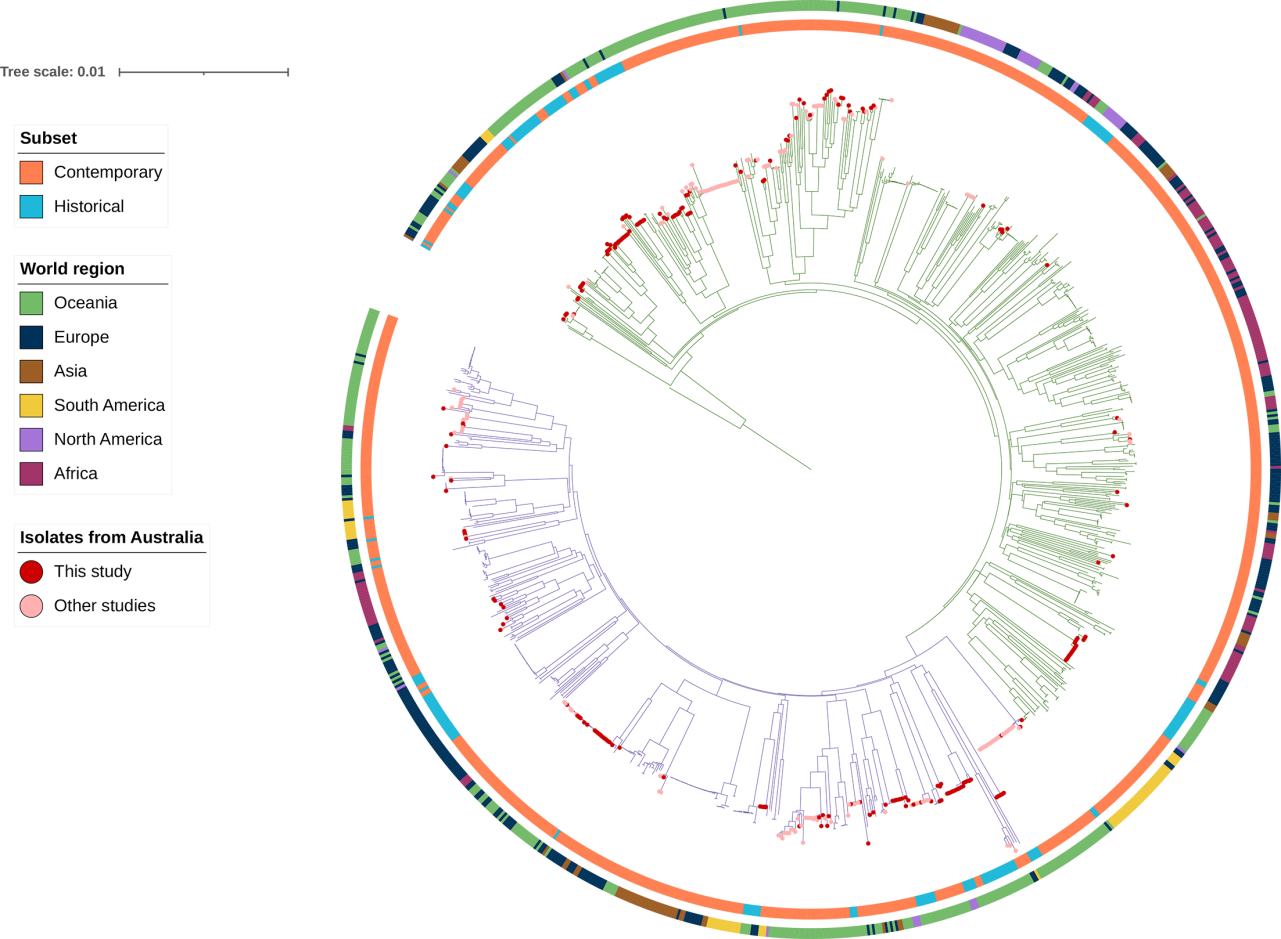
Phylogenetic tree (mashtree) of 1,095 *C. diphtheriae* isolates from six continents. The root was set based on the divergence point of *C. belfantii* FRC0043a (not shown), and the branches of the main phylogenetic lineages, Mitis and Gravis, are coloured in green and purple, respectively. The inner ring around the tree corresponds to contemporary isolates (2004–2023, orange) and historical isolates (prior to 2004, blue). The outer ring is coloured by continent where each isolate was reported. Symbols at the branch tips represent Australian *C. diphtheriae* isolates (*n*=384), Victorian isolates from this study (red) (*n*=210) and Australian isolates from other studies (pink) (*n*=174).

Analysis of the global phylogenetic structure of *C. diphtheriae* revealed that isolates from Victoria, Australia (this study), were highly diverse and dispersed across the global phylogeny (Fig. 2). *C. diphtheriae* isolates from other Australian studies were similarly dispersed across the global phylogeny, indicating that Australian isolates are phylogenetically diverse and belong to multiple lineages disseminated worldwide (Fig. 2).

### Phenotypic AMR was only present in contemporary strains

**Fig. 3.**
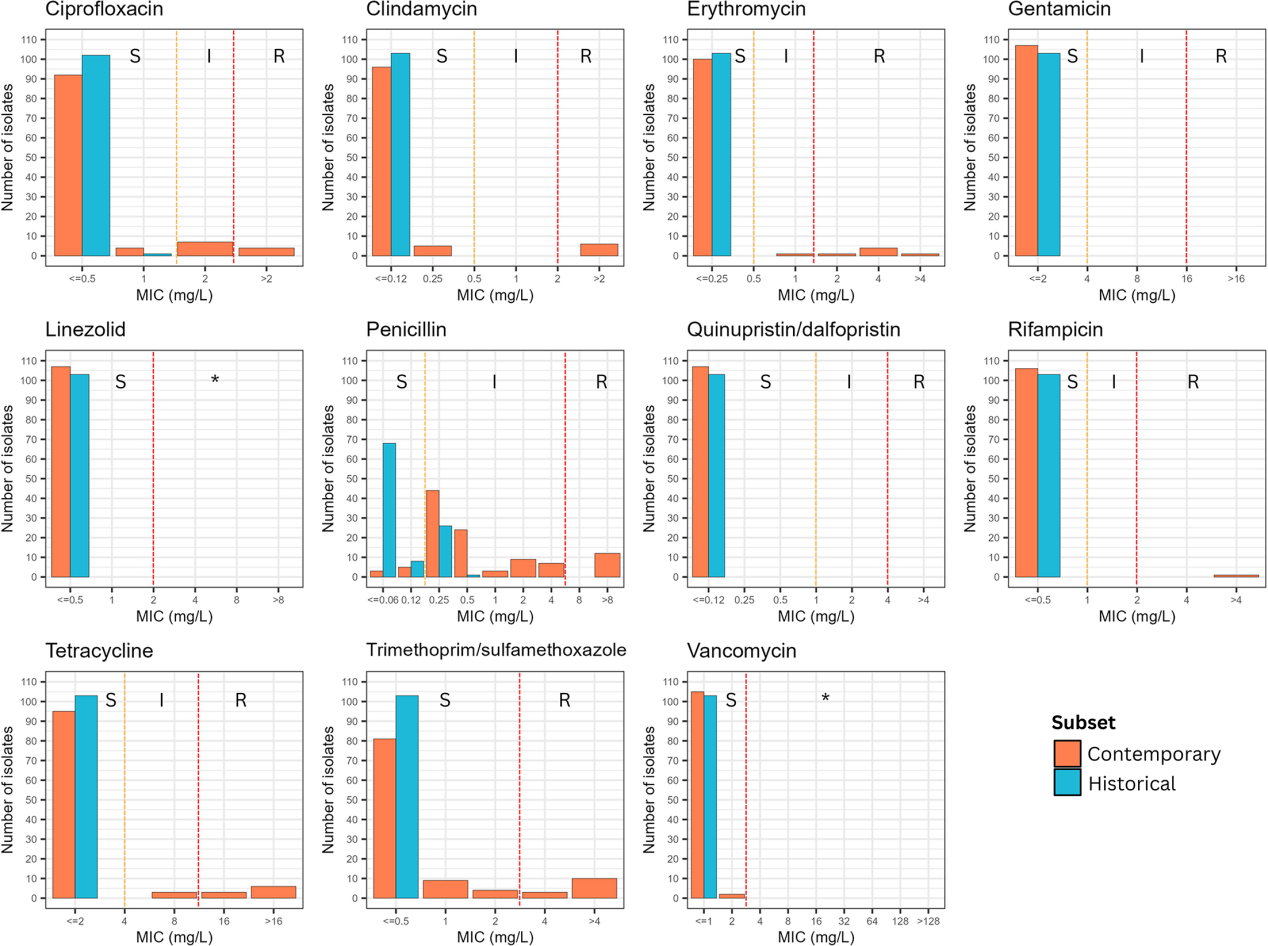
Antimicrobial susceptibility of *C. diphtheriae* isolates. Distribution of MIC values of 11 antimicrobials obtained from BMD for contemporary (*n*=107) and historical (*n*=103) isolates. Colour of the bars represents contemporary (orange) and historical (blue) isolates of *C. diphtheriae*. MIC values were interpreted as susceptible (S), intermediate (I) or resistant (R) following the CLSI M45 (third ed.) guidelines. Asterisk (*) denotes ‘non-susceptible’ category as per CLSI M45 guidelines (2015), where absence or rare occurrence of resistant strains is reported for this organism/antimicrobial agent combination.

AST of all 210 *C*. *diphtheriae* isolates revealed susceptibility profiles for 11 antimicrobials, including the first-line antimicrobials: penicillin and erythromycin. All historical isolates (*n*=103) were susceptible to 10/11 antimicrobial agents: ciprofloxacin, clindamycin, erythromycin, gentamicin, linezolid, quinupristin/dalfopristin, rifampicin, tetracycline, trimethoprim/sulfamethoxazole and vancomycin. The majority of historical isolates (*n*=76; 73.8%) were susceptible to penicillin, while a quarter of the subset (*n*=27; 26.2%) tested as intermediate to penicillin, as per CLSI breakpoints [36] for *Corynebacterium* spp. No penicillin-resistant phenotypes were observed in historical isolates (Fig. 3 and Table S3).

All contemporary isolates (*n*=107) were susceptible to 4/11 antimicrobial agents: gentamicin, linezolid, quinupristin/dalfopristin and vancomycin. A small number of contemporary isolates (7.5%; *n*=8) were fully susceptible to all 11 antimicrobial agents. Resistant phenotypes were observed for 7/11 antimicrobial agents: ciprofloxacin, clindamycin, erythromycin, penicillin, rifampicin, trimethoprim/sulfamethoxazole and tetracycline (Fig. 3 and Table S3). Phenotypic resistance to at least one antimicrobial agent was observed in 30.8% (*n*=33) of contemporary isolates. For penicillin, only 7.5% (*n*=8) of contemporary isolates tested as susceptible, while 74.8% (*n*=80) and 17.7% (*n*=19) tested as intermediate and resistant, respectively. For erythromycin, 93.4% (*n*=100) contemporary isolates tested as susceptible, while 0.9% (*n*=1) and 5.6% (*n*=6) tested as intermediate and resistant, respectively (Table S3).

Notably, eight contemporary isolates were identified as MDR *C. diphtheriae*. Five MDR isolates were resistant to both first-line antimicrobials, penicillin and erythromycin, while the remaining three isolates were not resistant to either of the first-line antimicrobials. All MDR isolates were non-toxigenic isolates from cutaneous samples, isolated between 2016 and 2023. Multidrug resistance was not observed among *tox*-positive isolates. However, two *tox*-positive contemporary isolates (of ST243) were resistant to tetracycline, and one was further resistant to trimethoprim/sulfamethoxazole (Table S3).

### Prevalence and associations of genetic AMR determinants and associated MGEs

**Fig. 4.**
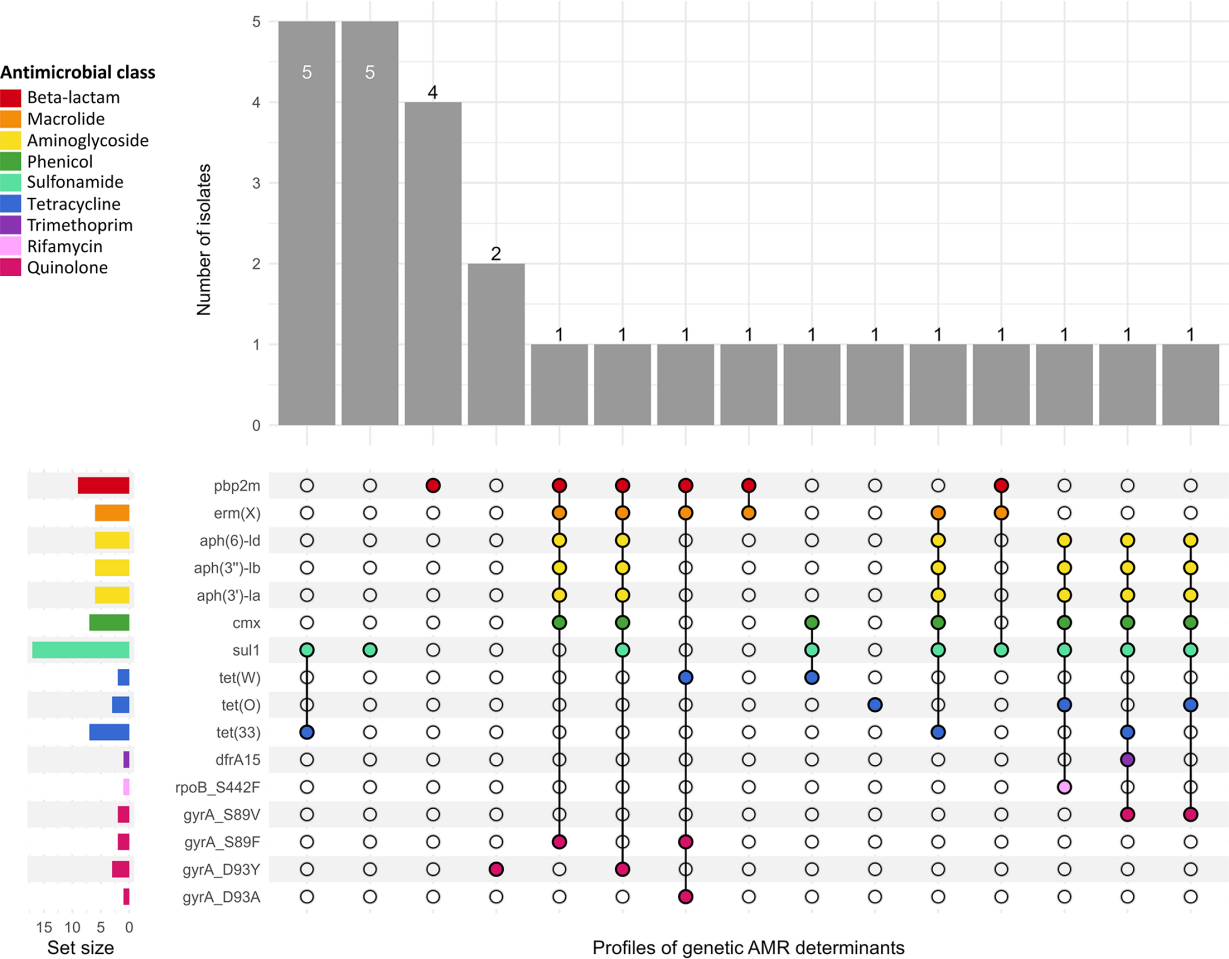
Upset plot of 27 contemporary *C. diphtheriae* isolates exhibiting different profiles of genetic AMR determinants. Genetic AMR determinants listed in the *y*-axis were detected by AMRFinderPlus (v3.12.8). *y*-axis bars (set size) demonstrate the total number of isolates with a particular AMR gene or mutation. *x*-axis bars represent number of isolates with a particular combination of AMR determinant/s. Colours of the set bars indicate the antimicrobial class (beta-lactam, macrolide, aminoglycoside, phenicol, sulfonamide, tetracycline, trimethoprim, rifamycin and quinolone) conferred by the corresponding gene/s or mutation/s.

A search for AMR genotypes revealed that there were no known AMR genes or mutations present in the historical *C. diphtheriae* isolates. However, 27 contemporary isolates carried at least one previously reported AMR determinant. These included antimicrobial genes conferring resistance to beta-lactams (*pbp2m*), macrolides [*erm*(*X*)], aminoglycosides [*aph*(*3′*)*-Ia*, *aph*(*6*)*-Id* and *aph*(*3″*)*-Ib*], tetracyclines [*tet*(*W*), *tet*(*33*) and *tet*(*O*)], phenicols (*cmx*), sulfonamides (*sul1*) and trimethoprim (*dfrA15*), as well as *C. diphtheriae-*specific point mutations in *gyrA* and *rpoB* conferring resistance to quinolones (*gyrA*_D93Y, *gyrA*_D93A, *gyrA*_S89F and *gyrA*_S89V) and rifamycin (*rpoB*_S442F), respectively (Fig. 4 and Table S3). A *bla*_OXA_ gene was observed in a single isolate but had an internal stop codon. Isolates carrying AMR genes were distributed across the Victorian *C. diphtheriae* phylogeny (Fig. S1).

A *pbp2m* gene was observed in nine isolates (all tested resistant or intermediate to penicillin), while *erm*(*X*) was observed in six isolates (all tested as resistant or intermediate to erythromycin). All isolates that tested as resistant or intermediate to tetracycline (*n*=12) carried one of the three identified tetracycline resistance genes [*tet*(*W*), *tet*(*33*) or *tet*(*O*)]. All three genes conferring resistance to aminoglycosides [*aph*(*3′*)*-Ia*, *aph*(*6*)*-Id* and *aph*(*3″*)*-Ib*] were always present together in six contemporary isolates. Phenotypically MDR isolates (*n*=8) were all *tox*-negative and carried a minimum of two and a maximum of eight known AMR determinants. All five MDR isolates with resistance to penicillin and erythromycin carried both *pbp2m* and *erm*(*X*) genes (Fig. 4 and Table S3).

AMR genes and mutations were rarely observed among *tox*-positive isolates, with only two *tox*-positive isolates (of ST243) carrying both *sul1* and *tet*(*33*) genes, consistent with their observed phenotypes (Table S3 and Fig. S1).

Across 13 isolates with AMR genes, multiple predicted plasmids and a single integron were found to harbour AMR genes (Table S4). The *erm*(*X*) gene was exclusively detected on predicted plasmid sequences in 6/6 isolates. In two of these isolates (AUSMDU00032758 and AUSMDU00078952), *erm*(*X*) was co-located with *pbp2m* on the predicted plasmid. A single class I integron was detected in one isolate, carrying *dfrA15*, *sul1* and *qacEΔ1* – a biocide tolerance gene. In the two *tox*-positive isolates (of ST243) carrying both *sul1* and *tet*(*O*), no plasmids or integrons were observed.

## Discussion

Genomic surveillance can provide a comprehensive understanding of a pathogen’s epidemiology and is increasingly being adopted for public health surveillance. In the context of a resurgence of *C. diphtheriae* globally, with the additional threat of AMR complicating treatment and containment [20,21, 23, 39], we sought to establish the basis for genomic epidemiologic surveillance for public health purposes in our location, where there has been a paucity of genomic data to date. Genomics-enabled public health surveillance of *C. diphtheriae* can support enhanced detection of transmission and monitoring trends for relevant pathogen characteristics, including *tox* carriage and AMR. This study investigated the population structure, *tox* carriage and AMR of 210 contemporary and historical isolates of *C. diphtheriae* in Victoria, Australia. We identified a highly diverse population structure of *C. diphtheriae*, similar to what has been described in other settings [17,27, 39]. Also, we observed that the contribution of homologous recombination was 7.5 times higher to *C. diphtheriae* diversification than mutation, consistent with previous studies [17,27, 31, 40]. Such extensive recombination enables the rapid acquisition of genetic material, including AMR genes, potentially enhancing adaptability and pathogenicity [17,31]. These findings highlight the importance of recombination-aware genomic surveillance to monitor emerging variants and inform public health responses.

The most significant virulence factor of *C. diphtheriae* is diphtheria toxin, a highly potent exotoxin mediated via the *tox* carried in some corynebacteriophages [1]. PCR methods and genomic approaches have been honed to predict the presence of the *tox* accurately. This study demonstrated complete concordance between genomic and PCR methods for detecting the toxin gene in contemporary isolates, providing confidence that genomic surveillance alone can adequately detect *tox* presence, at least in our setting. Phenotypic expression of the toxin gene was not assessed using the Elek test [41] in this study, as the required antitoxin is not available in Australia. However, genomic analysis revealed no evidence of non-functional toxin genes, suggesting the absence of NTTB strains in this dataset. Our phylogenetic analysis revealed that *tox*-positive isolates were dispersed in multiple unrelated sublineages, suggesting independent acquisition of the toxin gene as observed in previous studies [17,27]. Virulence factors of *C. diphtheriae* other than the toxin gene were not investigated in this study. While reported in previous studies [14,22, 39], their direct contribution to clinical disease remains unclear and warrants further investigation.

This study identified a predominantly *tox*-positive population among historical *C. diphtheriae* strains and a predominantly *tox*-negative population in contemporary strains in Victoria, resulting in part due to an unavoidable sampling bias between the two datasets. During the collection timeframe of the historical isolates, *C. diphtheriae* detection was generally limited to cases presenting with classic diphtheria, and *tox*-negative strains would have been unlikely to be identified. In contrast, modern bacterial diagnostic methods such as MALDI-TOF MS have enhanced the detection of both *tox*-positive and *tox*-negative *C. diphtheriae* across a wider range of clinical presentations [13,14]. Furthermore, the observed pattern also mirrors the drastic decline of diphtheria cases in Victoria following widespread immunization [2,3]. This suggests an evolutionary trajectory away from toxin production, potentially driven by vaccine-induced host immunity, altered transmission dynamics and reduced fitness of toxigenic strains in a predominantly immunized population [42]. Although not investigated in this study, the dynamics of *tox*-encoding corynebacteriophages should be explored in future research to better understand *tox* diversity. This study observed both *tox*-positive and *tox*-negative isolates within the same ST – an evolutionary pattern reported in previous studies [17,27, 39]. Interestingly, both *tox*-positive and *tox*-negative historical isolates within ST1128 were closely related (<37 SNPs), suggesting toxin gene loss and/or gain events were potentially occurring around suspected transmission events (Fig. S2). Although, with small isolated numbers and limited metadata for historic isolates, this hypothesis could not be explored further. These findings emphasize the importance of incorporating historical *C. diphtheriae* genomes into genomic analyses, as they could provide crucial information on strain turnover and shifts in lineage distribution in the population. Such insights are critical for understanding pathogen population dynamics over time and for guiding ongoing genomic surveillance efforts.

Recent re-emergence of diphtheria has posed a public health burden to some countries, especially in African and European regions [24,43,46]. Comparatively, diphtheria outbreaks are extremely rare in Australia and no diphtheria outbreaks have been reported in Victoria in the last two decades. The most recent outbreak of *tox*-positive *C. diphtheriae* (of ST381) occurred in North Queensland, Australia, in 2022 [5], with 24 locally acquired cases reported between January and August. Most isolates (*n*=18) were from cutaneous samples, while six were respiratory, although only two patients exhibited classic respiratory diphtheria symptoms. No mortalities were reported. There was no evidence of transmission to Victoria, and ST381 was not observed among Victorian isolates in this study. However, in the event of re-emergence of diphtheria in Australia, genomics will play a critical role in identifying likely transmission routes, distinguishing multiple importations from occult local transmission. Through this study, we have established the genomic baseline and methods to allow these data to be rapidly produced and analysed, generating actionable data for public health responses.

Analysis of *tox*-negative contemporary isolates in this study revealed a high prevalence of ST32, which were epidemiologically not related. Apart from Australia, ST32 has been reported in European countries [14,27, 39]. *C. diphtheriae* strains of ST32 were observed to have enhanced virulence due to increased adhesion potential [14], possibly contributing to the relative frequency in the Australian population. ST32 was reported in the state of Victoria (this study) and New South Wales [14], but ST39 has been frequently reported among *tox*-negative isolates in North Queensland [5], demonstrating some genetic diversity of non-toxigenic *C. diphtheriae* strains between geographic locations within Australia.

This study identified genomically related *C. diphtheriae* strains, within the 0–100 pairwise SNPs distribution range, in both contemporary and historical subsets, with higher frequency observed among *tox*-positive historical isolates. The overrepresentation in the historical subset likely corresponds to diphtheria outbreaks in Victoria between 1950 and 1970, when transmission was not uncommon and immunization coverage against diphtheria was suboptimal [2,4]. However, suspected outbreaks could not be confirmed due to the limited available metadata. Notably, the repeated occurrence of closely related strains of ST32 between 2008 and 2023 indicates the sustained presence of this ST within the contemporary population structure in Victoria, reflecting a persistent clone rather than isolated outbreak events. While this study employed MLST to examine the population structure of *C. diphtheriae* in Victoria across historical and contemporary subsets covering distinct temporal ranges, alternative approaches such as core genome MLST would be valuable for investigating outbreak scenarios, as previously described [24,27, 45].

Emergence of AMR in *C. diphtheriae* poses a significant challenge to the clinical management of *C. diphtheriae* infections, as well as to control pathogen transmission. Unfortunately, clinical susceptibility breakpoints lack standardization between the two dominant susceptibility testing guidelines, European Committee on Antimicrobial Susceptibility Testing (EUCAST) and CLSI, posing challenges in detection and comparison of AMR in a global context [17,39]. Some studies have proposed implementing tentative epidemiological cut-off (TECOFF) values for antimicrobials [17,47], with a recent study [48] allowing EUCAST to develop evidence-based breakpoints for *C. diphtheriae*. However, the CLSI [36] and EUCAST (https://www.eucast.org/clinical_breakpoints) guidelines specify different conditions for AST of *C. diphtheriae* by BMD. In this study, we followed the CLSI protocol [36], because of the two methods, only the media required for the CLSI protocol is commercially available in Australia. Accordingly, MICs were interpreted following the CLSI breakpoints for *Corynebacterium* spp. [36] to ensure standardized reporting. Whilst discordances between clinical breakpoints remain, this study at least provides some baseline AMR data for interpretation and to potentially guide empiric therapy.

While previous studies have compared resistant phenotypes to genetic determinants of AMR in *C. diphtheriae*, very few Australian isolates were investigated in such studies [17,21, 49]. In this study, we provide a comprehensive overview of both antimicrobial-resistant phenotypes and known genetic determinants of AMR in *C. diphtheriae* isolates from Victoria, Australia. Our analysis of phenotypic resistance against first-line antimicrobials demonstrated an emerging resistance to penicillin (17.7%) and erythromycin (5.6%) in contemporary isolates compared to no resistance to both penicillin and erythromycin among historical isolates. Despite following different susceptibility testing guidelines, increased penicillin resistance or reduced susceptibility rates have been reported in multiple studies [17,49, 50]. In contrast, phenotypic resistance or reduced susceptibility to erythromycin remains relatively low [17,24, 50]. Phylogenetic distribution of AMR genes showed their presence across multiple unrelated sublineages (Fig. S1), indicating independent acquisition via horizontal gene transfer, consistent with previous studies [17,39]. Previous studies have identified MGEs – plasmids and integrons carrying AMR genes in *C. diphtheriae* [17,25, 26], including the MDR conjugative plasmid pLRPD with *pbp2m*, *erm*(*X*) and multiple other AMR genes [17]. In this study, plasmids were predicted from short-read genome assemblies, which cannot consistently resolve full plasmid assemblies. Therefore, the number and type of plasmid-associated AMR genes reported here may be incomplete. The class I integron detected in one isolate, carrying *dfrA15*, *sul1* and *qacEΔ1* genes, exhibited a typical structure consistent with previously reported class I integrons [17,26]. The identification of two isolates with predicted plasmids carrying both *pbp2m* and *erm*(*X*) in this study is a notable public health concern, highlighting the potential for simultaneous resistance to both first-line antimicrobials, penicillin and erythromycin, and the need for ongoing surveillance. While recent studies have significantly advanced our understanding of genomic determinants of AMR in *C. diphtheriae*, resistance mechanisms for several antimicrobials, including penicillin and erythromycin, have not been fully characterized, emphasizing the need for further research.

In conclusion, we have used comprehensive genomic analysis to demonstrate a highly diverse population structure in Victorian *C. diphtheriae* in both historical (predominantly *tox*-positive) and contemporary (predominantly *tox*-negative) strains, establishing a baseline for future investigations. The Victorian sequences are dispersed across the global *C. diphtheriae* phylogeny, reflecting multiple independent importations in the contemporary setting. We have demonstrated high concordance between PCR and *in silico* methods for the detection of toxin gene and shown the added value of genomic AMR detection to augment traditional phenotypic AST methods. Given the potential for global re-emergence of diphtheria, particularly in areas of conflict and low vaccine coverage, and with the added complication of increasing AMR, it is critical for public health and clinical laboratories in all settings to prepare for future epidemics of *C. diphtheriae*. As we have demonstrated, genomic surveillance likely plays a significant role in identifying transmission routes and patterns to inform public health actions. Future work should focus on providing equitable access to genomic and phenotypic surveillance across all countries, including low- and middle-income countries, enabling effective global surveillance and coordinated responses.

## Supplementary material

10.1099/mgen.0.001517 Uncited Supplementary Material 1.

10.1099/mgen.0.001517 Uncited Supplementary Material 2.

## Abbreviations

AMR: antimicrobial resistance
ANI: average nucleotide identity
AST: antimicrobial susceptibility testing
BMD: broth microdilution
CLSI: Clinical and Laboratory Standards Institute
EUCAST: European Committee on Antimicrobial Susceptibility Testing
iTOL: Interactive Tree of Life
MALDI-TOF MS: matrix-assisted laser desorption/ionization time-of-flight mass spectrometry
MDR: multidrug-resistant
MDU PHL: Microbiological Diagnostic Unit Public Health Laboratory
MGEs: mobile genetic elements
MIC: minimum inhibitory concentration
ML: maximum likelihood
MLST: multi-locus sequence type
NTTB: non-toxigenic toxin gene-bearing
ST: sequence type
*tox*: diphtheria toxin gene

## References

[1] Sharma NC, Efstratiou A, Mokrousov I, Mutreja A, Das B (2019). Diphtheria. Nat Rev Dis Primers.

[2] Gidding HF, Burgess MA, Kempe AE (2001). A short history of vaccination in Australia. Med J Aust.

[3] Winkler NE, Dey A, Quinn HE, Pourmarzi D, Lambert S (2022). Australian vaccine preventable disease epidemiological review series: diphtheria 1999–2019. Commun Dis Intell.

[4] Hall R (1917). Notifiable diseases surveillance, 1917 to 1991. Commun Dis Intell.

[5] Graham RMA, Rathnayake IU, Sandhu S, Bhandari M, Taunton C (2023). Genomic analysis of an outbreak of toxin gene bearing *Corynebacterium diphtheriae* in Northern Queensland, Australia reveals high level of genetic similarity. Epidemiol Infect.

[6] Barakett V, Morel G, Lesage D, Petit JC (1993). Septic arthritis due to a nontoxigenic strain of *Corynebacterium diphtheriae* subspecies mitis. Clin Infect Dis.

[7] de Santis A, Siciliano RF, Sampaio RO, Akamine M, Veronese ET (2020). Non-toxigenic *Corynebacterium diphtheriae* infective endocarditis with embolic events: a case report. BMC Infect Dis.

[8] Shanmugam L, Priyadarshi K, Kumaresan M, Sivaradjy M, Upadhyay P (2021). A rare case report of non-toxigenic *Corynebacterium diphtheriae* bloodstream infection in an uncontrolled diabetic with peripheral vascular disease. Cureus.

[9] Hogg GG, Strachan JE, Huayi L, Beaton SA, Robinson PM (1996). Non-toxigenic *Corynebacterium diphtheriae* biovar gravis: evidence for an invasive clone in a south-eastern Australian community. Med J Aust.

[10] Berry I, Bampoe V, Cole M, Ju H, McCall IC (2025). P-1318. Epidemiology and trends of *Corynebacterium diphtheriae* in the United States, 2016-2023. Open Forum Infect Dis.

[11] Zasada AA, Baczewska-Rej M, Wardak S (2010). An increase in non-toxigenic *Corynebacterium diphtheriae* infections in Poland--molecular epidemiology and antimicrobial susceptibility of strains isolated from past outbreaks and those currently circulating in Poland. Int J Infect Dis.

[12] Xie AG, Yomogida K, Berry I, Briggs NL, Esie P (2024). Notes from the field: increase in nontoxigenic *Corynebacterium diphtheriae* - Washington, 2018-2023. MMWR Morb Mortal Wkly Rep.

[13] Bernard KA, Pacheco AL, Burdz T, Wiebe D (2019). Increase in detection of *Corynebacterium diphtheriae* in Canada: 2006-2019. Can Commun Dis Rep.

[14] Timms VJ, Nguyen T, Crighton T, Yuen M, Sintchenko V (2018). Genome-wide comparison of *Corynebacterium diphtheriae* isolates from Australia identifies differences in the pan-genomes between respiratory and cutaneous strains. BMC Genomics.

[15] Doyle CJ, Mazins A, Graham RMA, Fang N-X, Smith HV (2017). Sequence analysis of toxin gene–bearing *Corynebacterium diphtheriae* strains, Australia. Emerg Infect Dis.

[16] Wołkowicz T, Zacharczuk K, Zasada AA (2023). Genomic analysis of *Corynebacterium diphtheriae* strains isolated in the years 2007–2022 with a report on the identification of the first non-toxigenic tox gene–bearing strain in Poland. Int J Mol Sci.

[17] Hennart M, Panunzi LG, Rodrigues C, Gaday Q, Baines SL (2020). Population genomics and antimicrobial resistance in *Corynebacterium diphtheriae*. Genome Med.

[18] Zakikhany K, Neal S, Efstratiou A (2014). Emergence and molecular characterisation of non-toxigenic tox gene-bearing *Corynebacterium diphtheriae* biovar mitis in the United Kingdom, 2003-2012. Euro Surveill.

[19] Hall E, Wodi AP, Kroger A, Hamborsky J, Wolfe C (2021). Epidemiology and prevention of vaccine-preventable diseases: public health foundation.

[20] Arcari G, Hennart M, Badell E, Brisse S (2023). Multidrug-resistant toxigenic *Corynebacterium diphtheriae* sublineage 453 with two novel resistance genomic islands. Microb Genom.

[21] Forde BM, Henderson A, Playford EG, Looke D, Henderson BC (2021). Fatal respiratory diphtheria caused by β-lactam–resistant *Corynebacterium diphtheriae*. Clin Infect Dis.

[22] Araújo MRB, Prates FD, Ramos JN, Sousa EG, Bokermann S (2024). Infection by a multidrug-resistant *Corynebacterium diphtheriae* strain: prediction of virulence factors, CRISPR-Cas system analysis, and structural implications of mutations conferring rifampin resistance. *Funct Integr Genomics*.

[23] Mina NV, Burdz T, Wiebe D, Rai JS, Rahim T (2011). Canada’s first case of a multidrug-resistant *Corynebacterium diphtheriae* strain, isolated from a skin abscess. J Clin Microbiol.

[24] Hoefer A, Seth-Smith H, Palma F, Schindler S, Freschi L (2025). *Corynebacterium diphtheriae* outbreak in migrant populations in Europe. N Engl J Med.

[25] Tauch A, Bischoff N, Brune I, Kalinowski J (2003). Insights into the genetic organization of the *Corynebacterium diphtheriae* erythromycin resistance plasmid pNG2 deduced from its complete nucleotide sequence. Plasmid.

[26] Barraud O, Badell E, Denis F, Guiso N, Ploy MC (2011). Antimicrobial drug resistance in *Corynebacterium diphtheriae* mitis. *Emerg Infect Dis*.

[27] Guglielmini J, Hennart M, Badell E, Toubiana J, Criscuolo A (2021). Genomic epidemiology and strain taxonomy of *Corynebacterium diphtheriae*. J Clin Microbiol.

[28] ABS (2024). National, state and territory population Canberra: Australian Bureau of Statistics. https://www.abs.gov.au/statistics/people/population/national-state-and-territory-population/latest-release.

[29] Souvorov A, Agarwala R, Lipman DJ (2018). SKESA: strategic k-mer extension for scrupulous assemblies. Genome Biol.

[30] Dazas M, Badell E, Carmi-Leroy A, Criscuolo A, Brisse S (2018). Taxonomic status of *Corynebacterium diphtheriae* biovar Belfanti and proposal of *Corynebacterium belfantii* sp. nov. Int J Syst Evol Microbiol.

[31] Bolt F, Cassiday P, Tondella ML, Dezoysa A, Efstratiou A (2010). Multilocus sequence typing identifies evidence for recombination and two distinct lineages of *Corynebacterium diphtheriae*. J Clin Microbiol.

[32] Pallen MJ (1991). Rapid screening for toxigenic *Corynebacterium diphtheriae* by the polymerase chain reaction. J Clin Pathol.

[33] Minh BQ, Schmidt HA, Chernomor O, Schrempf D, Woodhams MD (2020). IQ-TREE 2: new models and efficient methods for phylogenetic inference in the genomic era. Mol Biol Evol.

[34] Didelot X, Wilson DJ (2015). ClonalFrameML: efficient inference of recombination in whole bacterial genomes. PLoS Comput Biol.

[35] Letunic I, Bork P (2024). Interactive Tree of Life (iTOL) v6: recent updates to the phylogenetic tree display and annotation tool. Nucleic Acids Res.

[36] CLSI (2016). Methods for Antimicrobial Dilution and Disk Susceptibility Testing of Infrequently Isolated or Fastidious Bacteria, 3rd ed.

[37] Robertson J, Nash JHE (2018). MOB-suite: software tools for clustering, reconstruction and typing of plasmids from draft assemblies. Microb Genom.

[38] Néron B, Littner E, Haudiquet M, Perrin A, Cury J (2022). IntegronFinder 2.0: identification and analysis of integrons across bacteria, with a focus on antibiotic resistance in *Klebsiella*. Microorganisms.

[39] Hennart M, Crestani C, Bridel S, Armatys N, Brémont S (2023). A global *Corynebacterium diphtheriae* genomic framework sheds light on current diphtheria reemergence. Peer Commun J.

[40] Grosse-Kock S, Kolodkina V, Schwalbe EC, Blom J, Burkovski A (2017). Genomic analysis of endemic clones of toxigenic and non-toxigenic *Corynebacterium diphtheriae* in Belarus during and after the major epidemic in 1990s. BMC Genomics.

[41] Engler KH, Glushkevich T, Mazurova IK, George RC, Efstratiou A (1997). A modified Elek test for detection of toxigenic corynebacteria in the diagnostic laboratory. J Clin Microbiol.

[42] Will RC, Ramamurthy T, Sharma NC, Veeraraghavan B, Sangal L (2021). Spatiotemporal persistence of multiple, diverse clades and toxins of *Corynebacterium diphtheriae*. Nat Commun.

[43] Badell E, Alharazi A, Criscuolo A, Almoayed KAA, Lefrancq N (2021). Ongoing diphtheria outbreak in Yemen: a cross-sectional and genomic epidemiology study. *Lancet Microbe*.

[44] Kofler J, Ramette A, Iseli P, Stauber L, Fichtner J (2022). Ongoing toxin-positive diphtheria outbreaks in a federal asylum centre in Switzerland, analysis July to September 2022. Euro Surveill.

[45] Badenschier F, Berger A, Dangel A, Sprenger A, Hobmaier B (2022). Outbreak of imported diphtheria with *Corynebacterium diphtheriae* among migrants arriving in Germany, 2022. Euro Surveill.

[46] Balakrishnan VS (2024). Diphtheria outbreak in Nigeria. *Lancet Microbe*.

[47] Marosevic DV, Berger A, Kahlmeter G, Payer SK, Hörmansdorfer S (2020). Antimicrobial susceptibility of *Corynebacterium diphtheriae* and Corynebacterium ulcerans in Germany 2011-17. J Antimicrob Chemother.

[48] Berger A, Badell E, Åhman J, Matuschek E, Zidane N (2024). *Corynebacterium diphtheriae* and *Corynebacterium ulcerans*: development of EUCAST methods and generation of data on which to determine breakpoints. J Antimicrob Chemother.

[49] Zou J, Chorlton SD, Romney MG, Payne M, Lawson T (2020). Phenotypic and genotypic correlates of penicillin susceptibility in nontoxigenic *Corynebacterium diphtheriae*, British Columbia, Canada, 2015–2018. Emerg Infect Dis.

[50] Benamrouche N, Hasnaoui S, Badell E, Guettou B, Lazri M (2016). Microbiological and molecular characterization of *Corynebacterium diphtheriae* isolated in Algeria between 1992 and 2015. Clin Microbiol Infect.

