# Supplementary material for: Population structure and antimicrobial resistance of Corynebacterium diphtheriae in Victoria, Australia
Source: Microb Genom. 2025 Oct 1;11(10):001517. doi: 10.1099/mgen.0.001517 (PMC12488388; doi:10.1099/mgen.0.001517)
Supplement: Uncited Supplementary Material 1. [file mgen-11-01517-s001.pdf]

Population structure and antimicrobial resistance of *Corynebacterium diphtheriae* in Victoria, Australia

Author names:

Lamali Sadeesh Kumar[1,2], Sarah L. Baines[1,3,4], Kylie Hui[2,3], Janet Strachan[5], Norelle L. Sherry[1,2,3,6], Benjamin P. Howden[1,2,3,4,6]

Affiliations:

[1] Department of Microbiology and Immunology, The University of Melbourne at the Peter Doherty Institute for Infection and Immunity, Melbourne, Victoria, Australia
[2] Microbiological Diagnostic Unit Public Health Laboratory, Department of Microbiology and Immunology, The University of Melbourne at the Peter Doherty Institute for Infection and Immunity, Melbourne, Victoria, Australia
[3] WHO Collaborating Centre for Antimicrobial Resistance, The Peter Doherty Institute for Infection and Immunity, Melbourne, Victoria, Australia
[4] Centre for Pathogen Genomics, The University of Melbourne, Melbourne, Victoria, Australia
[5] Communicable Diseases, Community and Public Health, Department of Health, Melbourne, Victoria, Australia
[6] Department of Infectious Diseases & Immunology, Austin Health, Heidelberg, Victoria, Australia

Supplementary Figures:

Supplementary Figure S1: Phylogenetic tree (maximum likelihood, recombination adjusted) of 210 *Corynebacterium diphtheriae* isolates from Victoria, Australia.

Supplementary Figure S2: Ridgeline plots of pairwise SNP distances within the ten most frequently reported sequence types (STs) among of 210 *Corynebacterium diphtheriae* isolates from Victoria, Australia.

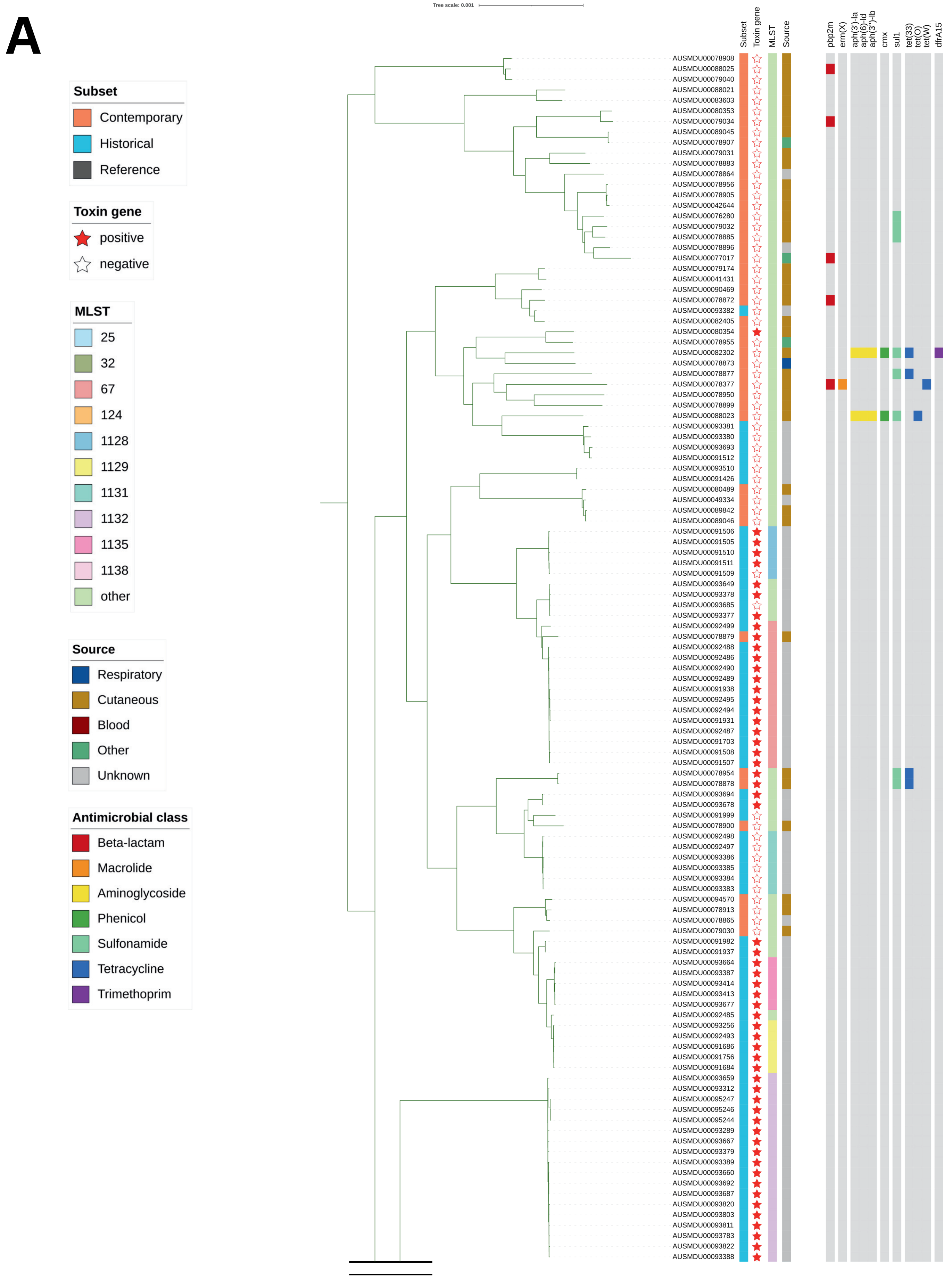

Supplementary Figure S1: Phylogenetic tree (maximum likelihood, recombination adjusted) of 210 *Corynebacterium diphtheriae* isolates from Victoria, Australia. A: Mitis lineage (Green). **B:** Gravis lineage (Purple). The tree is constructed from a whole genome alignment, relative to the reference genome *C. diphtheriae* NCTC 13129 (NC_002935.2). The root was set based on the divergence point of *Corynebacterium belfantii* FRC0043a (not shown). The first column (left side) aligned against the tree corresponds to contemporary (n=107, orange) and historical (n=103, blue) isolates, and reference strain (grey). The second column denotes the presence (filled star) or absence (unfilled star) of diphtheria toxin gene (tox). The third column represents the ten most common sequence types (STs) and all remaining STs grouped as 'Other STs'. The fourth column represents the source of the isolates; respiratory, cutaneous, blood, other and unknown. The last 11 columns (right side) represent antimicrobial resistance (AMR) genes observed in the dataset and the colours represent the antimicrobial class that the respective gene/s conferring resistance to.

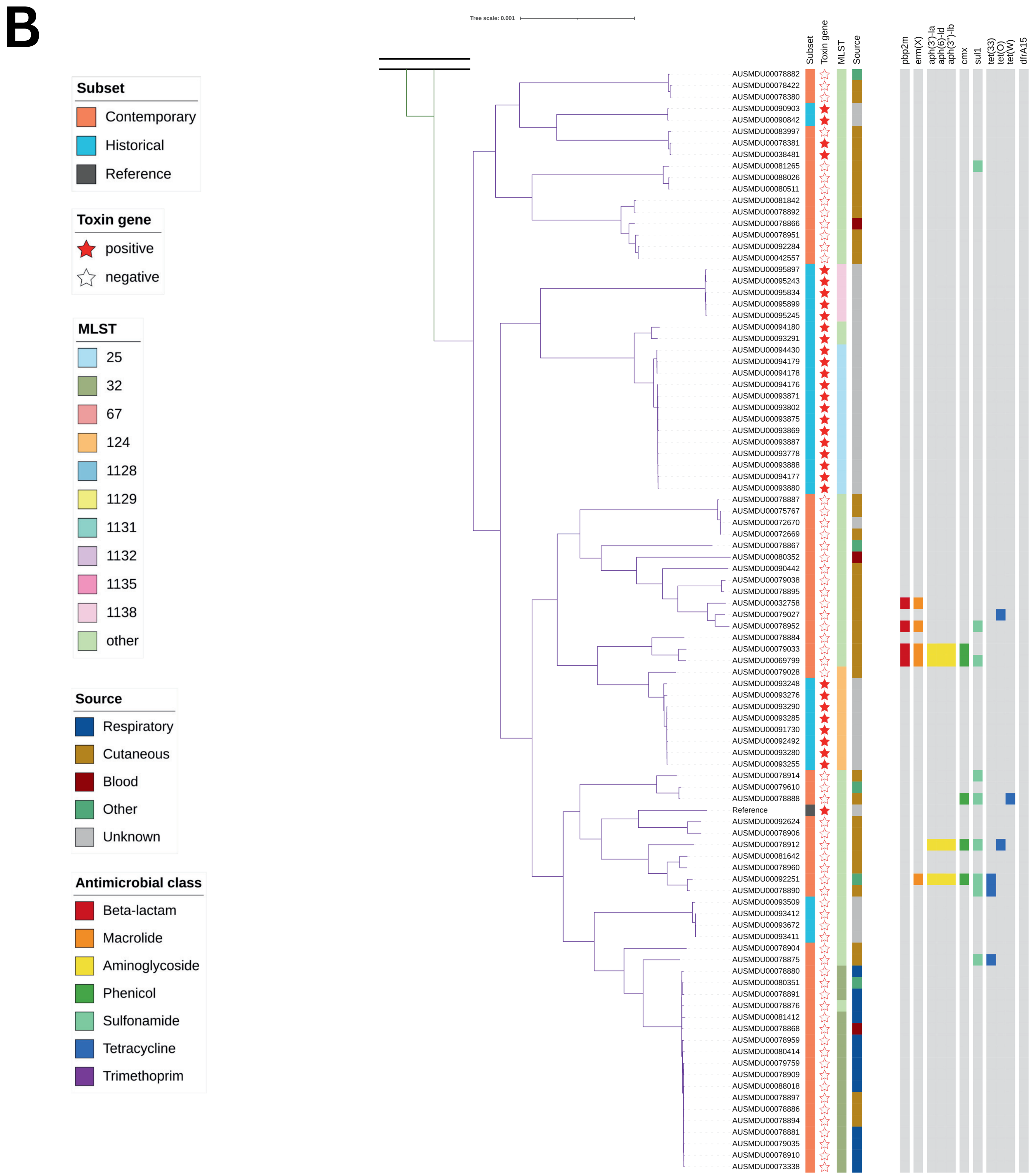

Supplementary Figure S1: Phylogenetic tree (maximum likelihood, recombination adjusted) of 210 *Corynebacterium diphtheriae* isolates from Victoria, Australia. A: Mitis lineage (Green). **B:** Gravis lineage (Purple). The tree is constructed from a whole genome alignment, relative to the reference genome *C. diphtheriae* NCTC 13129 (NC_002935.2). The root was set based on the divergence point of *Corynebacterium belfantii* FRC0043a (not shown). The first column (left side) aligned against the tree corresponds to contemporary (n=107, orange) and historical (n=103, blue) isolates, and reference strain (grey). The second column denotes the presence (filled star) or absence (unfilled star) of diphtheria toxin gene (tox). The third column represents the ten most common sequence types (STs) and all remaining STs grouped as 'Other STs'. The fourth column represents the source of the isolates; respiratory, cutaneous, blood, other and unknown. The last 11 columns (right side) represent antimicrobial resistance (AMR) genes observed in the dataset and the colours represent the antimicrobial class that the respective gene/s conferring resistance to.

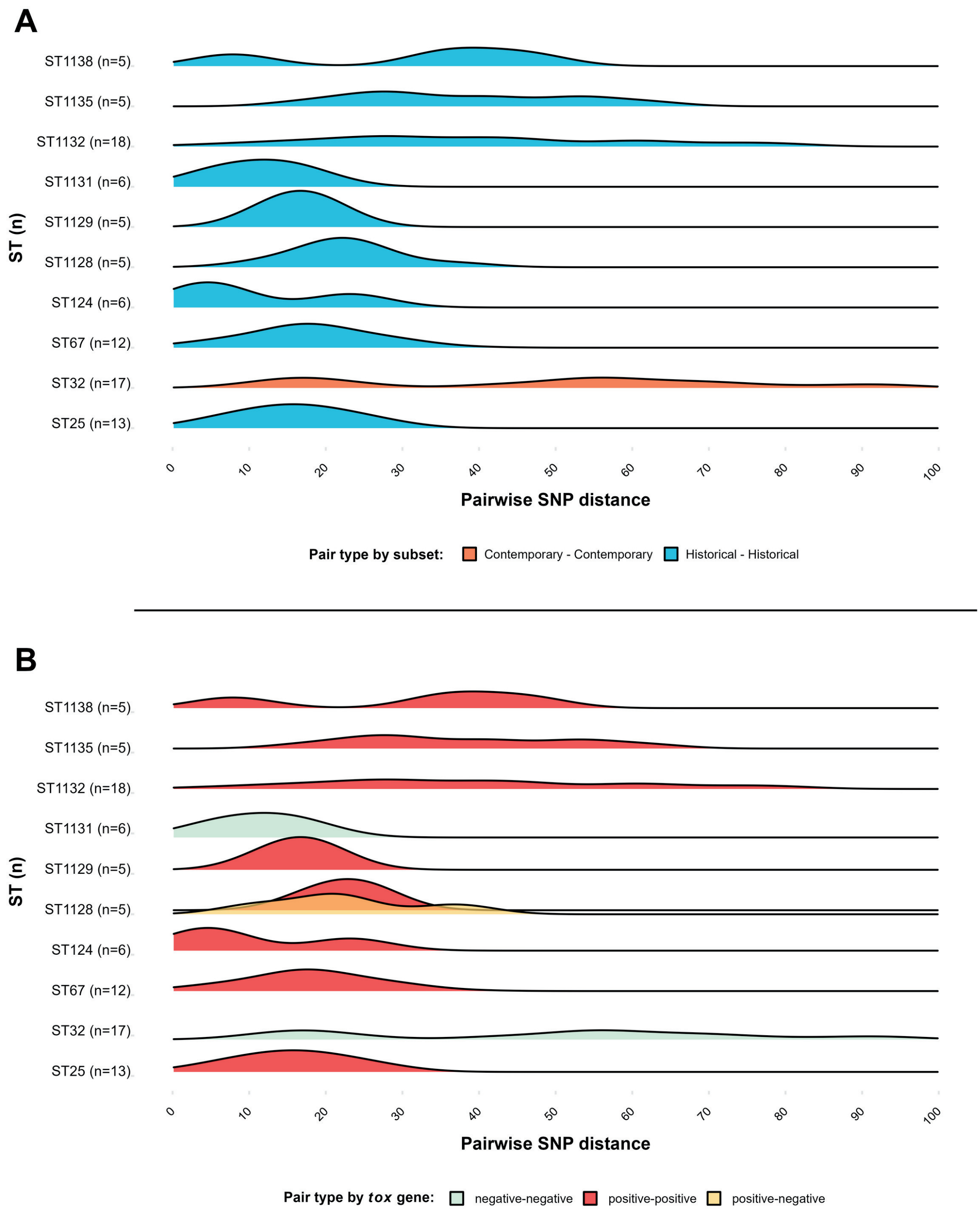

Supplementary Figure S2: Ridgeline plots of pairwise SNP distances within the ten most frequently reported sequence types (STs) among of 210 *Corynebacterium diphtheriae* isolates from Victoria, Australia. A – ridge colours indicate temporal subset (historical–historical, contemporary–contemporary). **B** – ridge colours indicate tox gene status (negative–negative, positive–positive, positive–negative). Only isolate pairs differing by <100 SNPs within each ST are included. Distances were calculated from the whole-genome SNP alignment relative to reference genome *C. diphtheriae* NCTC 13129, excluding positions missing in one or both isolates. x-axis: pairwise SNP distance; y-axis: sequence type (ST) and number of isolates (n) included for each ST.
